# Zn Modification of Pd/TiO_2_/Ti Catalyst for CO Oxidation

**DOI:** 10.3390/ma16031216

**Published:** 2023-01-31

**Authors:** Payam Samadi, Michal J. Binczarski, Waldemar Maniukiewicz, Aleksandra Pawlaczyk, Jacek Rogowski, Elzbieta Szubiakiewicz, Malgorzata I. Szynkowska-Jozwik, Izabela A. Witonska

**Affiliations:** Institute of General and Ecological Chemistry, Lodz University of Technology, 116 Zeromskiego Street, 90-924 Lodz, Poland

**Keywords:** Pd-Zn/TiO_2_/Ti catalysts, PEO, CO oxidation, porous layer TiO_2_ on Ti, Pd catalysts

## Abstract

The main goal of this study was to modify the activity of Pd/TiO_2_/Ti catalyst in the reaction of CO oxidation by the addition of Zn. Plasma electrolytic oxidation (PEO) of Ti wire was conducted to produce a uniform porous layer of TiO_2_. A mixture of Pd and Zn was then introduced by means of adsorption. After reduction treatment, the activity of the samples was examined by oxidation of 5% CO in a temperature range from 80–350 °C. Model catalysts with sufficient amounts of the metals for physico-chemical investigation were prepared to further investigate the reaction between Pd and Zn during CO oxidation. The structures and compositions of the samples were investigated using scanning electron microscopy with energy dispersive spectroscopy (SEM-EDS), time-of-flight secondary ion mass spectrometry (TOF-SIMS), inductively coupled plasma mass spectrometry (ICP-MS), Fourier transform infrared (FTIR), and X-ray diffraction (XRD). Modification of Pd/TiO_2_/Ti catalyst by Zn with a Pd:Zn atomic ratio of 2:1 decreased the temperature of complete CO oxidation from 220 °C for Pd/TiO_2_/Ti to 180 °C for Pd-Zn/TiO_2_/Ti. The temperature of 50% CO conversion on Pd-Zn(2:1)/TiO_2_/Ti was around 55 °C lower than in the reaction on monometallic Pd catalyst. The addition of Zn to the Pd catalyst lowered the binding energy of CO on the surface and improved the dissociative adsorption of oxygen, facilitating the oxidation of CO. FTIR showed that the bridging form of adsorbed CO is preferred on bimetallic systems. Analysis of the surface compositions of the samples (SEM-EDS, TOF-SIMS) showed higher amounts of oxygen on the bimetallic systems.

## 1. Introduction

Over 90% of industrial processes use solid catalysts, especially heterogenous metallic catalysts. One of the reactions that has received the most attention in the field of heterogeneous catalysis is CO oxidation [1,2,3]. It is very important for metallic systems to use carriers with highly porous structures, well-defined shapes with a well-developed surface area, good metal dispersion, and proper mechanical properties. From the industrial point of view the best supports are monolithic structures (metallic or ceramic), since these demonstrate better mechanical stability, heat resistivity, higher corrosion resistance, and lower flow resistance to reagents.

One of the techniques that can be used to produce a porous structure on a metallic carrier, such as Ti, is plasma electrolytic oxidation (PEO) [4,5,6]. During the PEO process, micro-arcs are produced on the surface, and a porous structure forms at the micro/nano scale. The PEO process can ensure a uniform porous layer on metal of any shape, which is important from an industrial point of view [7,8,9]. Metallic supported catalysts prepared by PEO can combine high thermal stability, corrosion resistivity, and low weight, making them suitable for use in the automobile industry as well as for purification of waste gases from stationary sources, which is important from the point of view of environmental protection. In contrast to monolithic ceramic-based catalysts, it is possible to produce metallic supported catalysts of different shapes and thicknesses, covered by a porous layer providing deposition sites for catalytic elements [10,11].

Intermetallic compounds have been found to be desirable for catalytic reactions. They include Pt-group metal catalysts, which have been studied for almost a century. According to the Langmuir–Hinshelwood (L–H) model, the possibility of CO oxidation is directly connected to the simultaneous presence of O_2_ and CO on the surface of the active site [12]. Adsorption and activation are frequently regarded as the rate-limiting steps in CO oxidation over metal-based catalysts. Increasing the availability of O_2_ on the catalyst surface can boost activity. Noble metals, such as Pd, Pt, and Au, supported on metallic carriers are very important catalysts in the CO oxidation process. A very important parameter determining the activity of the system in the oxidation reaction is size of the noble metal crystallites, which determines the dispersion of the metal on the surface. It has been shown that the activity of Au/TiO_2_ in the CO oxidation reaction can be increased by one order of magnitude by decreasing the size of the Au crystallites from 4 nm to 2 nm. The activity of Pt/SiO_2_ slightly increases when the size of Pt crystallites is increased from 1 nm to 5 nm. To modify the catalytic activity of these systems, a second metal can be added, which results in the formation of intermetallic compounds or solid solutions, in which various forms of interaction occur between the metallic components [13].

Different types of alloying systems have been investigated for enhancing catalytic activity, by increasing the dispersion of the active phase and producing new intermetallic active centers. Adding Cu to palladium can lower the intensity bands corresponding to bridge species in the hydrogeneration of furfural [14]. Adding Pb to palladium catalysts drastically improves the selectivity of Pd–Pb catalyst for the direct production of methyl methacrylate from methacrolein [15]. Karski et al. showed that the addition of small amounts of Bi (1–3 wt.%) to Pd catalyst has a positive influence on the catalytic properties of Pd–Bi/SiO_2_ systems in the oxidation of lactose and glucose [16,17]. The addition of a small amount of Ag in 5% Pd/SiO_2_ to silver can modify the intensity of the hydrogen evolution peak. The negative peak become less intense, suggesting that ß-PdH formation is inhibited. This phenomenon, caused by the formation of solid solutions of palladium and silver, may affect the rate of the hydrogen transfer reaction in the process of glucose oxidation [18]. In diesel, Pd–Pt bimetallic catalysts are more active than either metal individually. The synergistic effect of Pd and Pt weakens the adsorption of CO, which is helpful for improving the activity of the catalysts in CO oxidation at lower temperatures [19]. Adding La species helps to modify the nucleation of Pd and results in smaller Pd clusters, which are less susceptible to poisoning by CO [20]. Scott et al. and Venezia et al. have shown that Pd–Au alloys are superior to pure palladium for CO oxidation. In the Pd–Au system, Au atoms tune Pd–Pd bonds in model bimetallic compounds via the ensemble effect, which enhances catalytic activity [21,22].

The addition of Zn to Pd catalysts can lower the binding energy of CO and reduce the possibility of Pd poisoning by CO, especially at low temperatures. It has been found that the activity of Pd–Zn systems is far better than the performance of metallic Pd. The higher activity of these catalysts in the CO oxidation reaction is explained by the weakening of the CO bond and the easier binding of O_2_ to Pd sites modified by Zn [23].

In this study, TiO_2_/Ti systems were prepared by the PEO method. Ti wire was coated with a porous structure of TiO_2_ on the surface. The main goal was to modify the activity of the Pd/TiO_2_/Ti catalyst in the reaction of CO oxidation by the addition of Zn. The catalytic activity of the samples was tested using 5% CO under synthetic air in a flow reactor with a solid bed of catalyst. To the best knowledge of the authors, there have been no previous studies investigating the modification of Pd/TiO_2_/Ti catalyst by the addition of Zn. The structure and composition of the Pd/TiO_2_/Ti, Zn/TiO_2_/Ti, and Pd-Zn/TiO_2_/Ti catalysts were investigated using SEM–EDS, TOF–SIMS, XRD, FTIR, and ICP-MS. Due to the limitations of the available surface testing methods, powder model catalysts with sufficient amounts of the metals (Pd and Zn) were produced and used in further studies of their physico-chemical properties.

## 2. Materials and Methods

### 2.1. Preparation of Catalysts

Commercial Ti, grade II, in the form of wire was prepared for use as a catalyst base material by the PEO method. More details about the preparation of samples by PEO are given in [5]. The resulting Ti wire covered with a layer of TiO_2_ was washed with deionized water, dried to constant weight on a moisture analyzer at 105 °C, and then placed in 20 mL of an aqueous solution of a mixture of PdCl_2_ (PdCl_2_, Avantor, Gliwice, Poland) and ZnCl_2_ (ZnCl_2_, Avantor, Gliwice, Poland), with a molar ratio of metals Pd^2+^/Zn^2+^ = 4:1, Pd^2+^/Zn^2+^ = 2:1, and Pd^2+^/Zn^2+^ = 1:1. The concentration of Pd^2+^ in all the bimetallic solutions was 0.2% by weight. Since both Pd and Zn metals were present in the solution, the reaction can be described as a simultaneous adsorption process. Once adsorption equilibrium was reached (after 2 h), the samples were removed from the solution, washed with deionized water, and dried on a moisture analyzer at 105 °C to constant weight. Samples of the catalyst precursors were then placed in a tube furnace and reduced with hydrogen gas at a temperature of 400 °C for 2 h. Finally, Pd-Zn/TiO_2_/Ti bimetallic catalysts were obtained.

### 2.2. Model Catalyst

Due to the very thin nature of the porous layer of TiO_2_ on the surface of the Ti wire, the amounts of Pd and Zn adsorbed on the surface were very low. Therefore, to investigate the interaction between Pd and Zn on the surface of the bimetallic catalyst, model powder catalysts were prepared with larger amounts of metallic elements. Commercial TiO_2_ powder (P25) with surface area of 48.7 m^2^/g was used as a support to prepare 5 wt.% Pd, 5%Pd–2.5 wt.% Zn, and 2.5 wt.% Zn catalysts by the wet impregnation method. Each sample was then reduced in H_2_ at 400 °C for 2 h. Subsequently, 0.5 g of each sample was placed in a reactor to test its catalytic activity.

### 2.3. Catalytic Activity Test

After drying the precursors of catalysts in air at 100 °C, they were submitted to reduction in H_2_ at 400 °C for 2 h. In the final stage, the catalytic activity of samples was examined in a mixture of 5% CO and 95% synthetic air (50 mL/min) at temperatures in the range of 80–350 °C. Using a quartz tube reactor with a mass flow controller, the catalytic activity of the samples was evaluated in the CO oxidation process. A digital heating system and temperature controller were installed inside the reactor to monitor the process precisely. The samples of catalysts in each test weighed about 0.5 g.

### 2.4. CO Sorption on Catalysts Measured by FTIR

Carbon monoxide adsorption measurements were carried out by infrared Fourier transform spectroscopy (FTIR) using a Nicolet 6700 FTIR spectrometer equipped with an MCT detector (Thermo Scientific) and a transmission cell. The number of scans selected was 64 and the spectral resolution was set at 4 cm^−1^. Before the measurements, the samples were reduced in situ under hydrogen flow at 100 °C for 0.5 h. Next, the samples were cooled to 40 °C in Ar. Adsorption was performed for 20 min. Subsequently, desorption was carried out in Ar, during which spectra were collected every 2 min. Gas flow was set each time as 10 mL/min, and sample mass was established as 20 mg.

### 2.5. XRD Analysis of the Catalyst Structure

Room temperature powder X-ray diffraction patterns were collected using a PANalytical X’Pert Pro MPD diffractometer in the Bragg–Brentano reflection geometry (Copper Kα1 radiation, λ = 1.54059 Å, 40 kV/30 mA). The patterns were acquired in the range of 5–90◦ 2θ in continuous scan mode with a 0.0167◦ step size and an exposure per step of 30 s. The samples were rotated during the measurements to reduce the effect of preferred orientation in the crystalline phase. Phase composition and crystallite size analyses were performed using the PANalytical High Score Plus software package (ver 4.9) and the International Centre for Diffraction Data (ICDD) powder diffraction file (PDF-2 ver. 2020) database of standard reference materials. The average crystallite sizes were determined by employing Scherrer’s formula (d = Kλ/βcos θ), where λ is the wavelength of the X-ray (CuKα), β is the integral breadth of a reflection (in radians) located at the 2θ Bragg angle, K is the shape factor (0.9), and θ is the angle of diffraction.

### 2.6. TOF-SIMS Analysis of Catalyst Surface

TOF-SIMS spectra of the catalysts were acquired using a TOF-SIMS IV secondary ion mass spectrometer (IONTOF GmbH, Münster, Germany) equipped with high mass resolution time-of-flight mass analyzer and Bi liquid metal ion gun. The high current bunched mode of primary 25 keV Bi_3_^+^ ion beam operation was used during the analysis. A pulse width of 1 ns, a repetition frequency of 10 kHz, and an average current of 0.2 pA were set for the primary ion beam. During spectra acquisition, a surface area of 100 × 100 µm^2^ was irradiated with 25 keV Bi_3_^+^ ions for 30 s.

### 2.7. Surface Study of Catalysts by SEM

The structure of the porous layer was investigated by scanning electron microscopy (SEM). A scanning electron microscope (SEM S-4700, Hitachi, Tokyo, Japan) was used, equipped with an energy dispersive spectrometer (EDS, Thermo-Noran Inc., Madison, WI, USA). Quantitative analysis of the composition of the surface layer was performed using a JEOL JSM-6610 LV (Jeol, Japan) with an EDS X-MAX 80 (Oxford Instruments, High Wycombe, UK) detector. The accelerating voltage was set to 20 kV. Spot size was drawn to obtain a proper signal in the EDS detector. After setting the electron beam parameters, the maps of elements were recorded. The chemical compositions of the samples were determined using the AZtec (v4.4) program delivered with the detector.

### 2.8. Characterization of the Catalyst Composition by ICP

The samples were kept in solutions with different concentrations of Pd and Zn. Direct analysis of the liquid samples after catalyst decomposition was performed by inductively coupled plasma mass spectroscopy (ICP–MS) using a Thermo Scientific X-Series instrument with a quadrupole analyzer (UK). The samples were additionally studied using an inductively coupled plasma optical emission spectroscope (ICP–OES) by Thermo Scientific (UK). An iCAP 7400 optical emission spectrometer was employed to additionally verify the data acquired by ICP–MS, since no proper certificate reference material was available. No significant variation in the results obtained by ICP-MS and ICP-OES was observed for either of the studied metals, with the exception of the low concentrations of Pd and Zn, which were in some cases below the detection limits of optical emission spectrometry. Detailed descriptions of the analysis of the chemical composition of the catalysts using ICP-MS and ICP-OES techniques and of the method of sample preparation are provided in the Supplementary Materials.

## 3. Results

**Figure 1:**
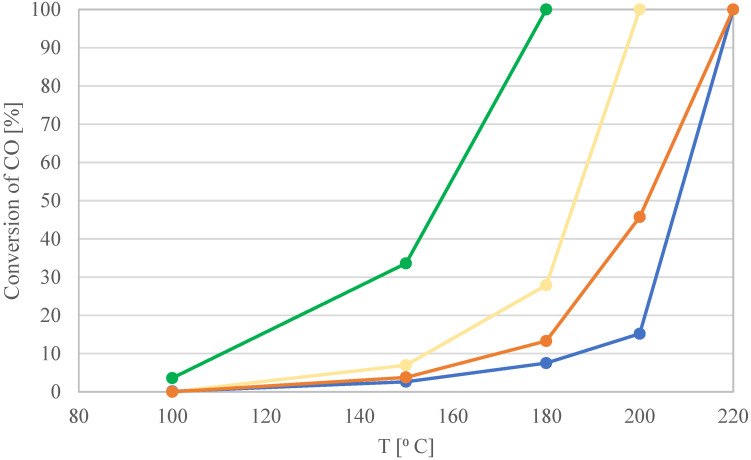
Catalytic activity of Pd and Pd–Zn/TiO_2_/Ti wire catalysts in different proportions: 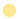 Pd/Zn = 4:1; 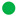 Pd/Zn = 2:1; 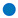 Pd/Zn = 1:1; 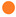 Pd.

In this study, we investigated the activity of zinc-modified palladium catalysts in the CO oxidation reaction. The activity of the catalysts was defined by the temperatures at which the degree of CO conversion was equal to 100%, 50%, or 10%. The T_100_, T_50_, and T_10_ of the wire Pd–Zn/TiO_2_/Ti samples are presented in Table 1. The catalytic activities of the wire samples are shown in Figure 1. Neither the TiO_2_/Ti nor Zn/TiO_2_/Ti catalysts were active in the temperature range from 25 °C to 350 °C.

The bimetallic Pd-Zn/TiO_2_/Ti catalyst prepared by co-adsorption of metals from a solution with a Pd:Zn molar ratio of 2:1 was characterized by the highest activity. For this catalyst, the CO conversion rate reached 100% at 175 °C. It can be concluded that the addition of an appropriate amount of Zn has a positive effect on the activity of the Pd/TiO2/Ti catalysts, shifting the temperature of complete oxidation of CO to CO_2_ from 220 °C to 175 °C.

It is well known that alloying metals on the surface of catalysts can increase the efficiency of the systems, by decreasing activation energy, and so reducing reaction temperatures. Alloying can also reduce the amount of noble metal in the active catalyst and lower total metal loading [24,25]. According to the literature, the addition of Zn lowers the temperature at which CO oxidation can be achieved, by lowering the binding energy of CO on the surface of the bimetallic active centers [19]. In other words, CO and O_2_ are adsorbed at the intermetallic active sites on the surface of bimetallic Pd–Zn catalysts much more easily than on the surface of monometallic systems. Due to the Langmuir–Hinshelwood (LH) mechanism, both reactants must be equally present on the surface to provide the best reactivity possible. At lower temperatures, there is a strong bond between Pd and CO, which prevents the simultaneous presence of oxygen on the surface. As a result, CO and O_2_ compete for available adsorption sites in CO/O_2_ mixes. Strong CO adsorption can result in thick CO layers, which significantly reduce the rate of O_2_ adsorption. As a result, there is very little CO oxidation activity, which is known as CO poisoning [12]. As can be seen in Figure 1, increasing the concentration of Zn in the mixed solution of 0.2 wt.% Pd–Zn to the molar ratio Pd:Zn = 2:1 increased the activity of the wire samples. However, a further increase (Pd:Zn = 1:1) resulted in lower activity.

**Figure 2:**
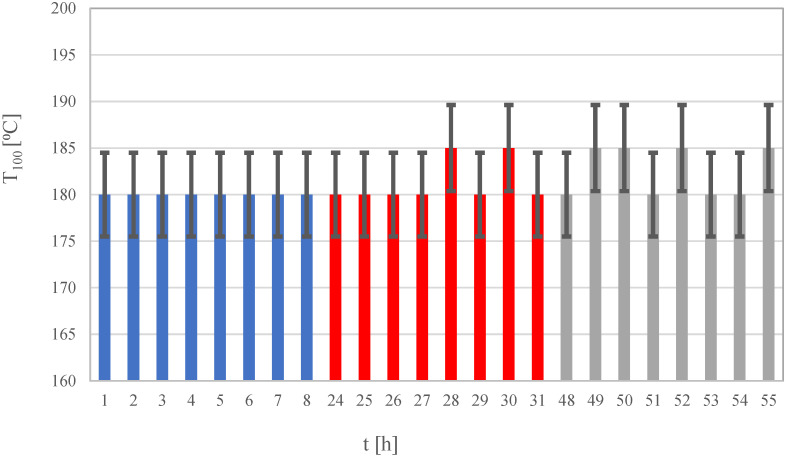
Activity of Pd-Zn/TiO_2_/Ti (Pd:Zn = 2:1) catalysts on subsequent days of oxidation reaction, expressed as T_100_.

Considering that the obtained bimetallic wire catalysts may be important in industrial applications due to their low metal loading and simple preparation, as well as good mechanical properties and simplicity of the preparation method, their stability in the CO oxidation reaction was investigated. For this purpose, a weighed sample of the wire catalyst was placed in a flow reactor, washed with Ar to remove air, reduced at 400 °C (2 h) in H_2_, cooled in Ar, and fed with a mixture of 5% CO in synthetic air. The temperature was increased from room temperature to 180 °C in which complete conversion of CO to CO_2_ was observed by chromatography measurements. It should be noted that 5% CO is much higher than the concentration used by other researchers (typically 0.1–1%). The catalyst sample was held at a temperature of 180 °C for another hour, at which point gas composition analysis was performed. If complete CO conversion was not observed, the temperature was increased by 5 °C, and another chromatogram was made. The base temperature was 180 °C for the whole experiment, which resulted in complete CO conversion of the fresh catalyst. Eight analyses were performed in the course of a day. Then, the sample was cooled in the reaction mixture to room temperature and left overnight without unsealing the reactor. The next day, without reduction in the catalyst, a regent mixture was added to the catalyst bed while heating to T_100_. Measurements were carried out for 8 h, recording T_100_. The procedure was repeated the following day. The test results are presented graphically in Figure 2. No significant changes in catalyst activity were observed during the three days.

The ICP–MS technique was used to determine the amount of Pd and Zn on the wire catalysts (Table 2). The results provided information about the general content of the elements in the prepared catalytic systems but not about the surface composition of the catalysts. It can be concluded that the addition of Zn^2+^ to the solution of Pd^2+^ increased the adsorption of Pd on the surface up to Pd:Zn = 2:1. A larger amount of Zn^2+^ caused a decrease in the amount of Pd on the surface. By contrast, increasing the concentration of Zn^2+^ led to a proportional increase in the number of adsorbed atoms on the surface of the TiO_2_/Ti system. To better understand the surface composition of the Pd–Zn/TiO_2_/Ti, SEM–EDS analysis was conducted.

The results of elemental analyses by SEM–EDS are presented in Table 3. As can be seen, the amount of Pd on the surface of the sample with the highest activity was higher than in the other samples. The highest percentage of oxygen was also present in this catalyst. The larger amount of Pd measured by SEM–EDS analysis compared to ICP–MS is due to the fact that the SEM–EDS technique is a form of surface analysis, whereas ICP–MS is a form of bulk analysis. Therefore, a larger amount of Pd was detected by SEM–EDS analysis than by ICP-MS analysis. The surface concentration of Zn increased in proportion to the increases in the concentration of Zn^2+^ in the solution used in the adsorption process for catalyst preparation.

When the amount of Zn was increased to Pd:Zn = 2:1, the activity of the catalyst increased not only at lower temperature but also at higher temperatures. This confirmed that the presence of Zn improved the activity of the catalyst, by weakening the bond between CO and Pd particles and by increasing dissociative oxygen adsorption on the surface of the catalyst. There are two possible reasons for the increase in the activity of the Pd–Zn bimetallic catalyst. The first is connected with better dispersion of Pd on the surface, which takes place as a result of the dilution of surface clusters of palladium atoms by zinc atoms. The second is connected with the formation of intermetallic compounds, which may be considered as new active centers for the oxidation of CO. To confirm these hypotheses, SEM-EDS, XRD, TOF-SIMS, and FTIR techniques available at Lodz University of Technology Institute of General and Ecological Chemistry were used. Given the shape of the Pd–Zn/TiO_2_/Ti wire samples, their roughness and very low load of wire catalysts with metals, we decided to prepare a model catalyst on TIO_2_ powder (P25), containing sufficient amounts of metals to enable reliable results (metal load > 5%-wag.). The adsorption technique used for the preparation resulted in wire catalysts containing very small amounts of metals (<0.2%-the metal, according to ICP-MS analysis). Therefore, to produce the model catalyst we used the wet impregnation method, with the same output salts. The obtained bimetallic Pd-Zn/TIO_2_ catalyst contained 5 wt.% of Pd and 2.5 wt.% of Zn, corresponding to a molar ratio of Pd:Zn = 2:1 in the final catalyst.

**Figure 3:**
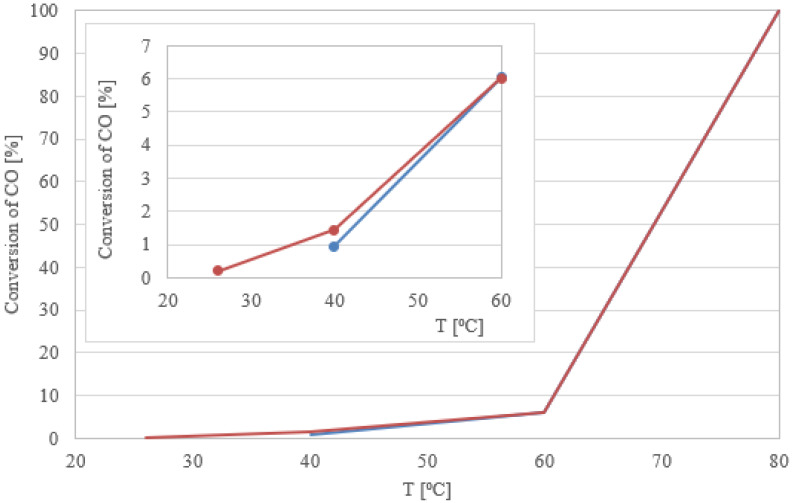
Catalytic activity of Pd/TiO_2_ and Pd–Zn/TiO_2_ (5 wt.% Pd; Pd/Zn = 2:1) of model catalysts.

First, we tested the activity of the palladium and palladium–zinc model catalysts in the CO oxidation reaction. For both the monometallic palladium catalyst and the bimetallic system, T_100_ was 80 °C. However, the promoting effect of Zn was visible at lower temperatures, where the conversion of CO was not complete. It was found that the reaction proceeded already at 25 °C (Figure 3).

**Figure 4:**
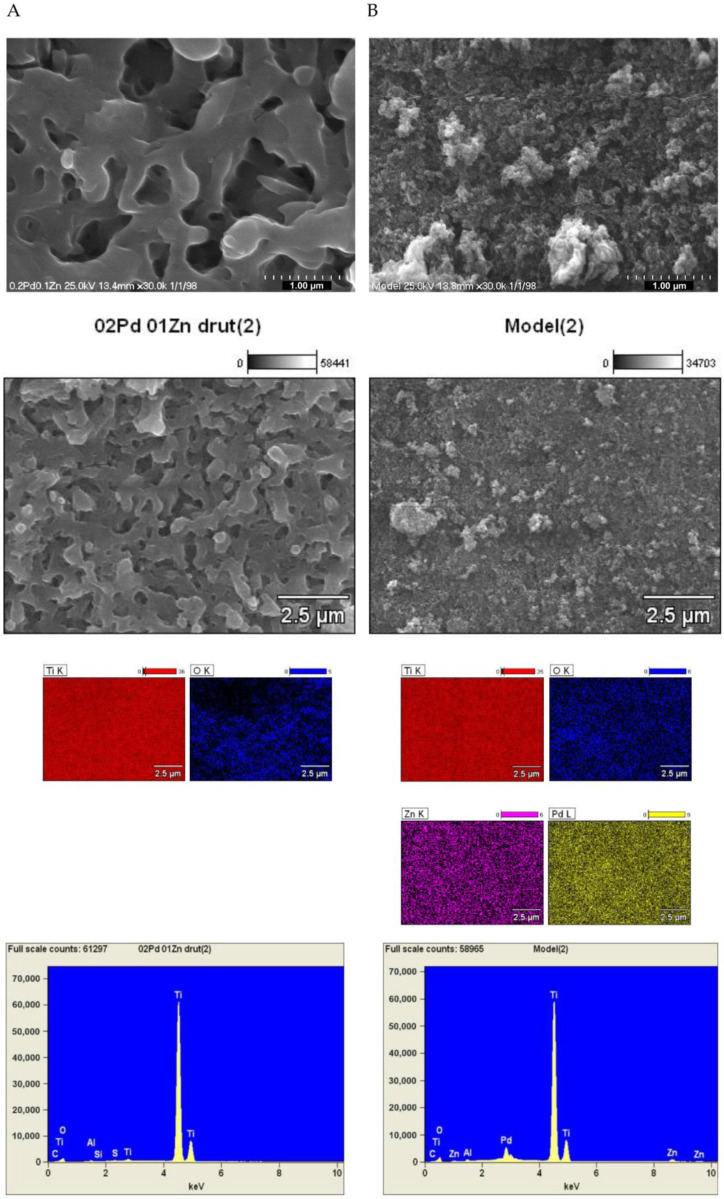
Pictures and analysis of elemental composition (maps) made using the SEM-EDS technique for (**A**) Pd-Zn/TiO_2_/Ti wire catalyst (Pd:Zn = 2:1) and (**B**) model Pd-Zn/TiO_2_ catalyst (Pd:Zn = 2:1).

Figure 4 shows SEM images and EDS maps collected for the wire and model catalyst, enabling comparison of their composition and surface morphology. In the case of wire catalysts, the metals on the surface are impossible to detect, due to the very small amounts introduced via the carrier pores by adsorption from the solution. In the case of the model catalyst, we can see an even distribution of palladium and zinc in the system.

To better understand CO interactions with the surface of Pd–Zn/TiO_2_/Ti catalysts, FTIR studies were performed. Due to the limitations of the FTIR technique [26], model powder mono- and bimetallic catalysts were used. The IR spectrum of CO adsorbed on the Pd surfaces exhibited two intense bands in the ranges 2100–2050 and 2000–1800 cm^−1^, which are typical for linear and bridged species of CO adsorbed on Pd [27].

**Figure 5:**
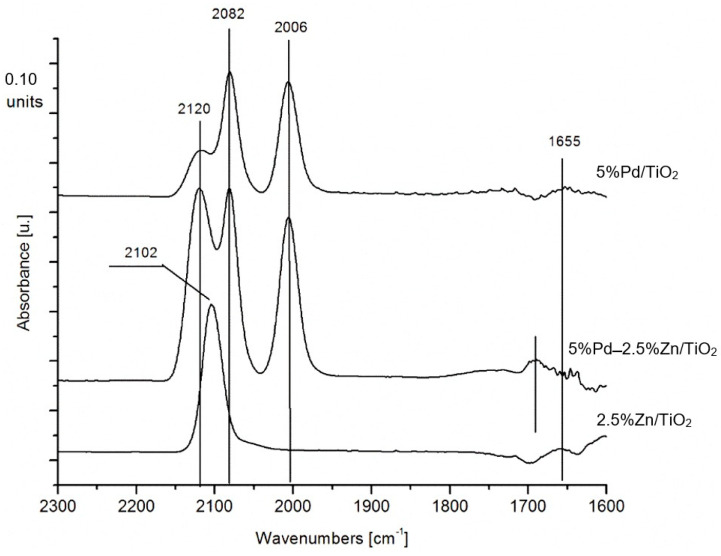
FTIR spectra of carbon monoxide adsorbed on bimetallic and monometallic catalysts, after reduction at 100 °C in H_2_ for 0.5 h, purging with Ar at 40 °C for 0.5 h, and CO sorption at 40 °C with flow of 10 mL·min^−1^. The IR spectra were collected at the same temperature after CO desorption in an Ar stream (30 mL·min^−1^) for 20 min.

The FTIR spectra of CO adsorbed at a low temperature (40 °C) over monometallic 5%Pd/TiO_2_, 2.5%Zn/TiO_2_, and bimetallic 5%Pd-2.5%Zn/TiO_2_ catalysts after reduction in H_2_ (100 °C, 0.5 h) are shown in Figure 5. In the spectrum for reduced monometallic 5%Pd/TiO_2_ catalyst, there are strong bands with a main maximum at 2006, 2082 cm^−1^ and a pronounced shoulder at 2120 cm^−1^. The first band is associated with the presence of the bridge-bonded CO species in the surface of Pd catalyst. The second is connected with linear adsorbed CO on the edges of Pd surface atoms and the linear adsorbed CO on the planes of Pd surface atoms. In the studied catalyst, the linear-bonded CO species in the surface of Pd are dominant. The amounts of bridge CO species increased when a small amount of Zn was incorporated in the Pd catalyst. In the spectrum of the 2.5%Zn/TiO_2_ catalyst, only one band at 2102 cm^−1^ can be observed, associated with CO adsorbed linearly on Zn atoms in the monometallic system.

**Figure 6:**
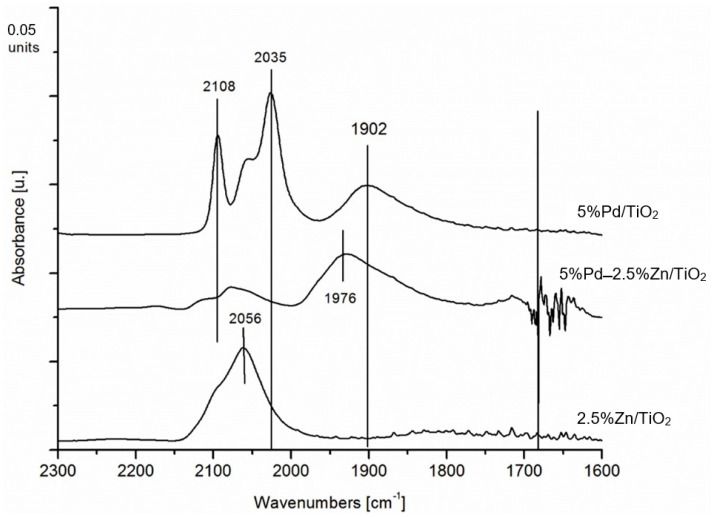
FTIR spectra of carbon monoxide adsorbed on bimetallic and monometallic catalysts after CO sorption at 40 °C with flow of 10 mL·min^−1^. The IR spectra were collected at the same temperature after CO desorption under an Ar stream (30 mL·min^−1^) for 20 min.

In Figure 6, similar bands in the range of 2000–2120 cm^−1^ can be seen as in Figure 5. In the case of the mono- and bimetallic palladium catalysts, a new band appears that can be attributed to bridge adsorption of CO on the Pd atoms located on the edges of the crystallites. There is a significant decrease in CO adsorption on the catalyst surface.

**Figure 7:**
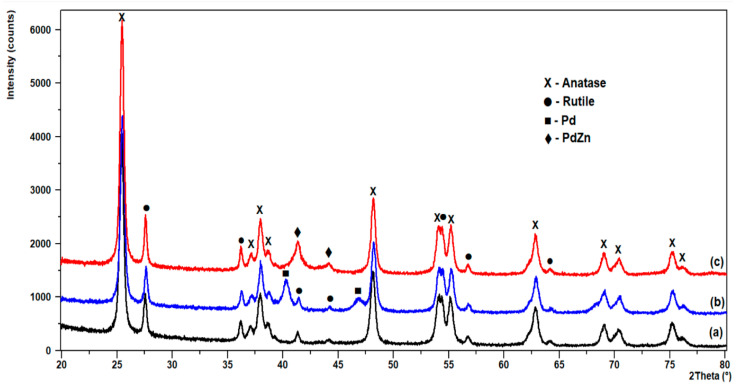
XRD patterns of model catalysts: (a) 2.5%Zn/TiO_2_; (b) 5%Pd/TiO_2_; (c) 2.5%Zn/5%Pd/TiO_2_.

The XRD patterns of the model samples are shown in Figure 7. Well-resolved reflection peaks in all patterns can be ascribed to the anatase (PDF-2 #021-1272) and rutile (PDF-2 #021-1276) phases of TiO_2_. In the case of the 2.5%Zn/TiO_2_ sample, no reflections from metallic zinc are visible. In the 5%Pd/TiO_2_ sample, peaks (111) and (200) for Pd appear at angles 2θ of 40.15° and 46.70°, respectively. The average Pd crystallite size estimated from these two peaks is 13 nm. For the sample of 2.5%Zn/5%Pd/TiO_2_, the Pd peaks disappear, while new peaks appear at 2θ angles of 41.30° and 43.81°. This indicates the formation of a PdZn intermetallic phase. Similar results for PdZn catalyst have been reported in previous studies [28,29]. The PdZn phase peaks overlap to some extent with the rutile peaks. The mean size of the PdZn crystallites after deconvolution was estimated at 9 nm.

**Figure 8:**
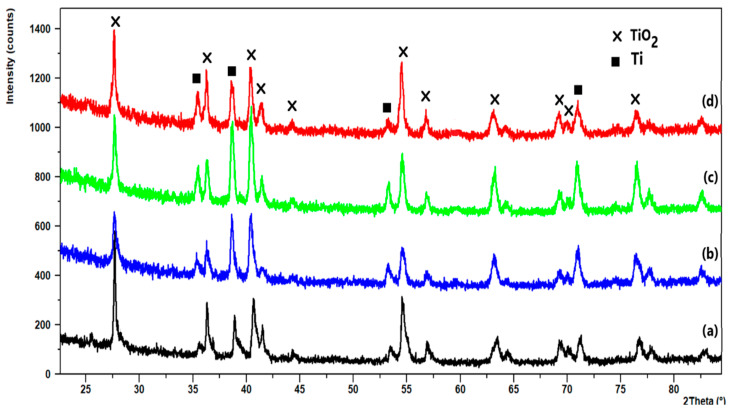
XRD patterns for Zn/TiO_2_ (a), Pd-Zn/TiO_2_ with Pd/Zn = 0.2/0.05 (b), Pd–Zn/TiO_2_ with Pd/Zn = 0.2/0.1 (c), and Pd-Zn/TiO_2_ with Pd/Zn = 0.2/0.2 (d).

Attempts to perform a similar analysis for wire samples containing much smaller amounts of Pd and Zn on Ti were unsuccessful (Figure 8). It was not possible to observe the PdZn phase, due to the limit of detection using the XRD method. The coating of these samples was therefore scratched off, to prepare a proper amount of material as a sample for investigation. However, as the amounts of palladium and zinc in the surface layer of the catalysts were also quite low, we were unable to unequivocally determine the phase composition of the samples using the XRD method. Moreover, peaks related to the active metals and any possible compositions, e.g., the PdZn intermetallic phase, overlapped strongly with the peaks derived from TiO_2_, making it difficult to identify the existing phases.

The XRD results obtained for the model catalyst confirmed the better dispersion of Pd in the bimetallic catalyst and the formation of an intermetallic phase, which may be the reason for the better activity of the Pd–Zn system for CO oxidation.

**Figure 9:**
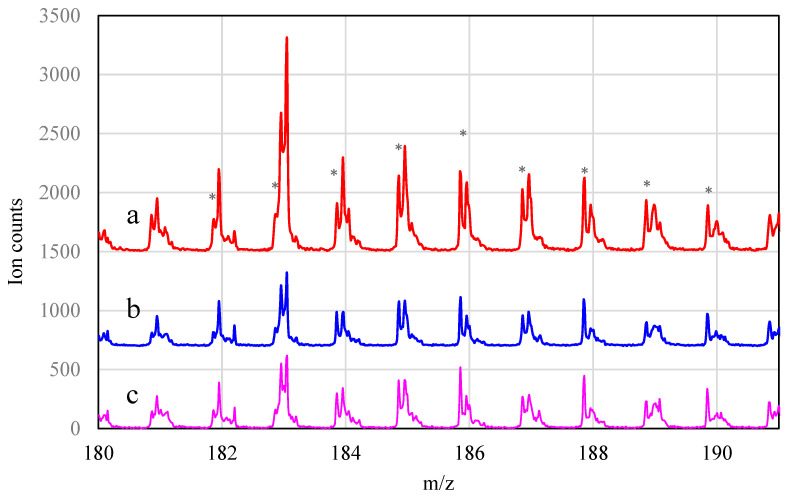
TOF–SIMS spectrum of the 5%Pd/TiO_2_ catalyst after impregnation (a), reduction (b), and reaction (c); * peaks assigned to PdTiO_2_^+^ ions in ToF-SIMS spectra.

The TOF–SIMS technique was used to study the interactions between the active metals and the catalyst support. Figure 9 presents the TOF-SIMS spectrum of the 5%Pd/TiO_2_. A characteristic isotopic pattern of PdTiO_2_^+^ ions can be resolved, despite the large number of peaks of other secondary ions. It can be assumed that the presence of PdTiO_2_^+^ ion peaks in the TOF–SIMS spectrum indicates the presence of Pd–TiO_2_ interaction in the catalyst.

**Figure 10:**
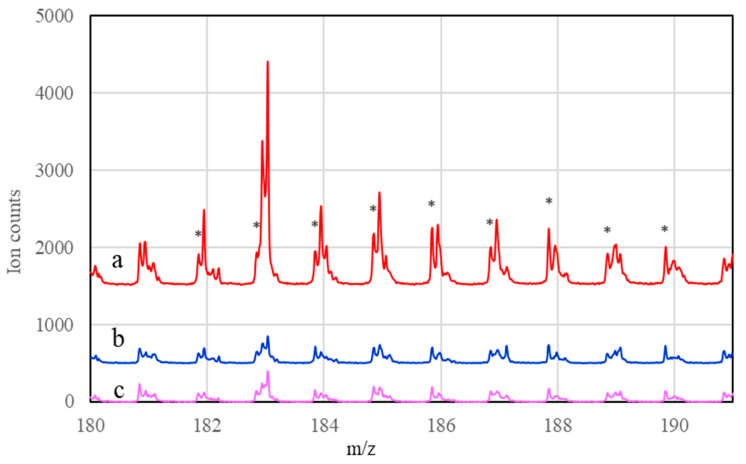
TOF–SIMS spectrum of the 2.5%Zn–5%Pd/TiO_2_ catalyst after impregnation (a), reduction (b), and reaction (c); * peaks assigned to PdTiO_2_^+^ ions in ToF-SIMS spectra.

Similarly, peaks for PdTiO_2_^+^ secondary ions were observed in the TOF–SIMS spectrum of the 2.5%Zn–5%Pd/TiO_2_ catalyst (Figure 10). This demonstrates that there was also an interaction between Pd and the support in the 2.5%Zn–5%Pd/TiO_2_ catalyst. For both catalysts, reduction results in a decrease in PdTiO_2_^+^ peak intensities. This may indicate that reduction in the catalyst leads to a partial decomposition of the compounds from which PdTiO_2_^+^ are formed. The TOF–SIMS spectra in Figure 9 and Figure 10 show that the catalyzed reaction does not affect the intensity of PdTiO_2_^+^ ions.

The Zn content in the wire catalysts was found to be too low for detection by TOF-SIMS. Therefore, the model catalyst with a higher Zn concentration was prepared. However, zinc has a very low emission of secondary ions and even in a model catalyst containing nominally 2.5 wt% Zn their emission is very low. In addition, the position of the Zn^+^ peak overlaps with the position of the intense peak from TiO^+^. Similarly, it is not possible to unequivocally determine the emission of PdZn^+^, because the peaks from this type of molecular ion coincide with the peaks from PdTiO^+^ with high intensity. Therefore, the TOF-SIMS spectra of the model catalyst with increased Zn concentration did not directly show a peak corresponding to molecular ions of PdZn^+^, which could be a proof of the interaction of the two metals in the studied Pd-Zn/TiO_2_ catalysts. The occurrence of such interactions can only be indirectly inferred from the effect of zinc content on palladium dispersion, as determined by the ratio of PdTiO_2_^+^/Pd^+^ (Table 4).

The TOF–SIMS results were also used to estimate differences in Pd dispersion in the catalyst. It was assumed that the catalysts with higher dispersion of Pd would be characterized by a larger Pd–TiO_2_ boundary region in relation to the surface area of the Pd crystallites, compared to the catalyst with lower dispersion. Therefore, in a catalyst with higher dispersion, a higher ratio of PdTiO_2_^+^ to Pd^+^ ion counts can also be expected. Table 4 presents the emission intensities of PdTiO_2_^+^ and Pd^+^ ions emitted from 5%Pd/TiO_2_ and 2.5%Zn-5%Pd/TiO_2_ catalysts.

**Figure 11:**
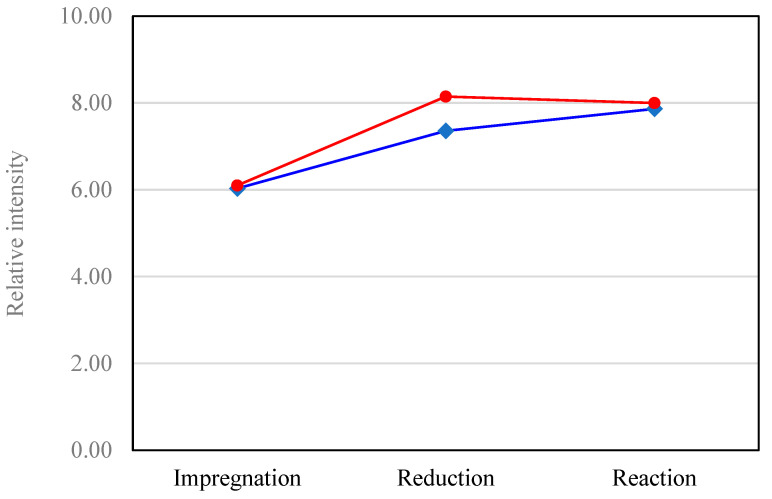
Relative values of PdTiO_2_^+^ to Pd^+^ ion emission for the 2.5%Zn–5%Pd/TiO_2_ [-●-] and 5%Pd/TiO_2_ catalysts [-♦-].

The relative numbers of PdTiO_2_^+^ to Pd^+^ ions given in Table 4 are additionally shown in Figure 11. The results suggest that the highest dispersion of palladium may be expected for 2.5%Zn–5%Pd/TiO_2_ catalysts after reduction.

To sum up, the catalyst surface testing techniques available at our institute allowed us to demonstrate the interactions between palladium and zinc only in the case of Pd–Zn/TiO_2_ (TiO_2_ type P25) model catalyst containing large amounts of metals. This preliminary research provided justifies the use of more advanced and expensive techniques, such as HR-TEM, XPS, Raman spectroscopy, or EXAFS, in future work. Such techniques have been successfully applied to the study of low Pt-loading Pt/TiO_2_ (TiO_2_ type P25) catalysts [30,31]. However, when selecting research techniques for the analysis of the surface composition of wire catalysts, it should be remembered that not only the low metal content but also the specific shape of the samples makes performing tests difficult.

## 4. Conclusions

In this study, the catalytic activity of Pd/TiO_2_/Ti metallic supported catalyst was improved by introducing Zn. A series of catalysts were prepared by PEO and adsorption methods, containing various amounts of Pd and Zn. The catalytic activities of the samples were measured. It was found that the addition of Zn, especially in the amount of Pd:Zn = 2:1, improved the activity of the catalyst and lowered the temperature of complete conversion of CO to CO_2_. The addition, Zn decreased the T50 of CO oxidation over Pd–Zn(2:1)/TiO_2_/Ti catalyst from 210 °C to 155 °C, by improving the dispersion of Pd and assisting the formation of PdZn intermetallic phase with new active centers. TOF–SIMS, XRD, SEM-EDS, ICP, and FTIR techniques were used to characterize the properties and surface structures of the catalysts. Due to the detection limits of some of these techniques and the low amounts of Pd and Zn on the TiO_2_/Ti support, model powder catalysts were prepared. The results not only proved the formation of PdZn intermetallic phases but also showed that the crystal size of Pd in the new PdZn phase reduced from 13 nm to 9 nm. Decreasing the reaction temperature could be very important for a variety of applications of catalysts, especially in the automobile industry.

## 5. Patents

On the basis of the data contained in the publication, patent application P. 443178 was developed and submitted to the Patent Office of the Republic of Poland on 19 December 2022.

## Figures and Tables

**Table 1:** T_100_, T_50_, and T_10_ for different wire catalysts.

Samples	T_100_ [°C]	T_50_ [°C]	T_10_ [°C] (<350)
TiO_2_/Ti	-	-	-
Pd/TiO_2_/Ti	220	210	180
Pd-Zn(4:1)/TiO_2_/Ti	200	185	155
Pd-Zn(2:1)/TiO_2_/Ti	180	155	110
Pd-Zn(1:1)/TiO_2_/Ti	220	210	185
Zn/TiO_2_/Ti	-	-	-

**Table 2:** ICP-MS analysis of the amounts of Pd and Zn in the wire catalysts.

Samples	wt.% Pd	wt.% Zn	Pd/Zn
Pd/TiO_2_/Ti	0.065	<DL *	<DL *
Pd-Zn(4:1)/TiO_2_/Ti	0.074	0.003	19.905
Pd-Zn(2:1)/TiO_2_/Ti	0.085	0.031	2.365
Pd-Zn(1:1)/TiO_2_/Ti	0.065	0.073	1.156

* DL—Detection Limit.

**Table 3:** SEM-EDS analysis of the amounts of Pd and Zn in wire catalysts [wt.%].

Samples	O	Ti	Zn	Pd
Pd/TiO_2_/Ti	44.15	55.68	-	0.09
Pd-Zn(4:1)/TiO_2_/Ti	39.02	60.74	0.03	0.08
Pd-Zn(2:1)/TiO_2_/Ti	47.92	51.96	0.03	0.1
Pd-Zn(1:1)/TiO_2_/Ti	38.9	60.91	0.05	0.04

**Table 4:** Secondary ion counts for 5%Pd/TiO_2_ and 2.5%Zn-5%Pd/TiO_2_ catalysts.

Catalyst	Process	TOF-SIMS Secondary Ion Counts		Relative Values of Ion Counts
		Pd^+^	PdTiO_2_^+^	PdTiO_2_^+^/Pd^+^
				10-2
5%Pd/TiO_2_	Impregnation	42,575	2567	6.03
	Reduction	14,053	1034	7.36
	Reaction	16,604	1306	7.86
2.5%Zn-5%Pd/TiO_2_	Impregnation	42,570	2596	6.10
	Reduction	8496	692	8.15
	Reaction	6902	552	8.00

## Data Availability

Data sharing is not applicable to this article.

## Supplementary Materials

The following supporting information can be downloaded at: https://www.mdpi.com/article/10.3390/ma16031216/s1, Table S1: Experimental parameters for ICP–OES and ICP–MS spectrometers.
