# Supplementary material for: Zn Modification of Pd/TiO2/Ti Catalyst for CO Oxidation
Source: Materials (Basel). 2023 Jan 31;16(3):1216. doi: 10.3390/ma16031216 (PMC9921276; doi:10.3390/ma16031216)
Supplement: Supplementary file 1 [file materials-16-01216-s001.zip › materials-2141172-supplementary.pdf]

Characterization of the catalyst composition by ICP

The catalyst material in the form of twisted Ti wire coated with Pd/Zn was divided into two separate samples. Each individual sample was weighed in a glass vial using an analytical balance. The approximate mass was about 100 mg. To decompose the catalyst layer on the Ti wire, 3 mL of concentrated nitric acid (BAKER ANALYZED, ultra-pure, USA) and 1 mL of concentrated hydrochloric acid (Chempur, ultra-pure, Poland) were added to each sample. Subsequently, the samples were placed in a closed pressure microwave system (UltraWAVE by Milestone, Italy). Blank tests containing the same reagents were also prepared and submitted to the same procedure. To guarantee the same conditions inside the stainless steel reactor of the pressure system, the samples were placed in a Teflon container filled with water. The reactor was then pumped with nitrogen gas under a pressure of 40 bar. After sample decomposition, the content of the vials was transferred into 100 mL flasks (class A) and diluted to the final volume of 100 mL with deionized water (Elix system, Millipore, Italy). The process of decomposition consisted of two steps, following the method described by Samadi et al. [5]:

- Stage I (20 min): programmed maximum pressure inside the reactor 120 bar, maximum microwave power 1500 W. Continuously increasing temperature inside the reactor up to 230°C.
- Stage II (10 min): programmed maximum pressure inside the reactor 130 bar, maximum microwave power 1500 W. Temperature of 230°C sustained for 10 min.

For calibration of both ICP spectrometers, single element palladium standard solution (Peak Performance, USA) and single element zinc solution (Merck, Germany) were used with subsequent dilution. As an internal standard, single element indium standard solution (by Merck, Germany) was added to each sample after decomposition and before fulfilling to a final volume with a deionized water. Satisfactory recovery values were achieved for both spectrometers.

All measurements were carried out for several isotopes of Pd and Zn (ICP–MS) and several emission lines of Pd and Zn (ICP–OES). The final results were calculated against the ^{105}Pb isotope and ^{66}Zn (ICP–MS), against the emission line of Pd at 340.458 nm in the axial position of the torch and at 213.856 nm in the axial position of the torch (ICP–OES). The signal for Zn and Pd was also monitored in the blank samples. However, no significant background increase connected with the use of the microwave oven system or chemical reagents was noticed. Real samples spiked with a known concentrations of Zn and Pd were used as a control material. The operating parameters of the spectrometers are listed in Table S1.

Table S1. Experimental parameters for ICP–OES and ICP–MS spectrometers.

Parameter	ICP-MS spectrometer	ICP-OES spectrometer
Instrument	X-Series, Thermo Scientific	iCAP 7400, Thermo Scientific
Detector	Simultaneous analogue/pulse counting electron multiplier	Solid-state CID86 chip
Optics/Analyzer	Chicane Ion Lens, quadrupole analyzer	Echelle type, 52.91 grooves/mm, simultaneous
RF generator frequency [MHz]	27.12	27.12
RF power (W)	1380	1150
Plasma gas flow [L/min]	12	12
Nebulizer gas flow [L/min]	Quartz concentric	Quartz concentric
Auxiliary gas flow [L/min]	0.8	0.5
Nebulizer	0.78	0.5
Spray chamber	Quartz impact bead	Quartz cyclonic
Number of replicates	3	3
Acquisition	Peak jumping, dwell time 20 ms, channel spacing 0.02, channels per mass 1	UV exposure time range 15 s, VIS exposure time range 5 s
Measurement range	2–255 amu	166–847 nm
Internal standard	^{115}In	In (I) 325.609 nm (Axial)
Analyte	^{105}Pd	Pd (I) 340.458 nm (Axial)
	^{66}Zn	Zn (I) 213.856 nm (Axial)
